# Fish in a Dish: Using Zebrafish in Authentic Science Research Experiences for Under-represented High School Students from West Virginia

**DOI:** 10.1089/zeb.2022.0074

**Published:** 2024-04-11

**Authors:** Rebecca A. Coltogirone, Summer L. Kuhn, Sean P. Freeland, Sadie A. Bergeron

**Affiliations:** ^1^ Department of Biology, West Virginia University, Morgantown, West Virginia, USA.; ^2^ Health Sciences Center & Health Sciences Technology Academy, West Virginia University, Morgantown, West Virginia, USA.; ^3^ Department of Neuroscience, West Virginia University, Morgantown, West Virginia, USA.

**Keywords:** zebrafish, science education, K-12, STEM, outreach

## Abstract

Early research experiences positively affect students' interest in STEM careers, and develop practical science and critical thinking skills. However, outreach opportunities are not equally accessible for all students. In states like West Virginia, where many students live in rural Appalachian communities, opportunities for engaging in STEM experiences are limited. In addition, rural teachers may not be equipped to provide authentic research experiences for students due to lack of resources or support. For many students in West Virginia, the Health Sciences and Technology Academy (HSTA) is a major opportunity for STEM engagement. Since its inception in 1998, HSTA has spread to 26 of 55 counties in West Virginia. The program recruits first-generation, low-socioeconomic status, rurally living, and African American high school students who are under-represented in STEM fields. Our research laboratory partnered with HSTA to implement an innovative, hands-on research camp using zebrafish for students participating in their annual junior-level biomedical sciences summer camp. Our camp was held in-person and adapted to an online format during the Covid-19 pandemic. We used pre–post surveys in both camps to assess impacts on science confidence and to collect information about general perceptions of zebrafish, research, and STEM fields. We found that students participating in the in-person and online camps experienced similar overall gains in science confidence. We also identified strong interest in zebrafish, research, and STEM degrees among online students. Online students did not prefer virtual learning experiences; however, they still enjoyed our camp. We also surveyed high school teachers volunteering for HSTA to identify factors that would encourage use of zebrafish in classrooms. The most prominent needs include classroom supplies, experience, and funding. Our successful science-education partnership demonstrates that zebrafish research experiences foster positive outcomes for under-represented students, and can inform future outreach efforts and collaborations with teachers.

## Introduction

Calls for advancing K-12 STEM education have inspired a wave of student-centered teaching practices aimed to satisfy the increasing demand for qualified STEM professionals in the United States.^1,2^ STEM outreach initiatives have grown significantly,^3^ however are not equally accessible to all students, with rural students being one of several disadvantaged groups. Rural students make up one-quarter of the U.S. public school enrollment, yet face challenges related to STEM education,^4^ such as limited access to extracurricular programs typically based in urban areas; less state funding for extracurricular activities, curriculum development, and research^5,6^; and lower recruitment of qualified STEM teachers compared with urban schools due to low financial incentive, remoteness, or inadequate facilities.^7–11^

In addition, studies suggest that rural parental values are at times incongruent with the need for enhanced STEM education, reducing incentive for schools to provide these opportunities.^6,12,13^ Together, these factors negatively impact students' science achievement and perception of their ability to succeed in STEM degrees and careers.^11,14,15^ Initiatives that provide hands-on STEM research experiences to rural and other disadvantaged and under-represented student groups could in part address these issues, and inspire new interest and confidence in STEM fields.

West Virginia lies in the heart of Appalachia, where 34 of 55 counties are rural and ∼38% of residents statewide reside in rural communities.^16,17^ The K-12 graduation rate in West Virginia exceeds the national average,^18^ yet there is a disconnect between high school graduation and college enrollment, as only 55% of graduates enroll in higher education institutions annually.^19^ Half of all graduates enroll in STEM degree programs, yet only 30% finish that original degree to completion.^20,21^ Thus, recruitment and retention in STEM programs have become a point of focus.

**FIG. 1.**
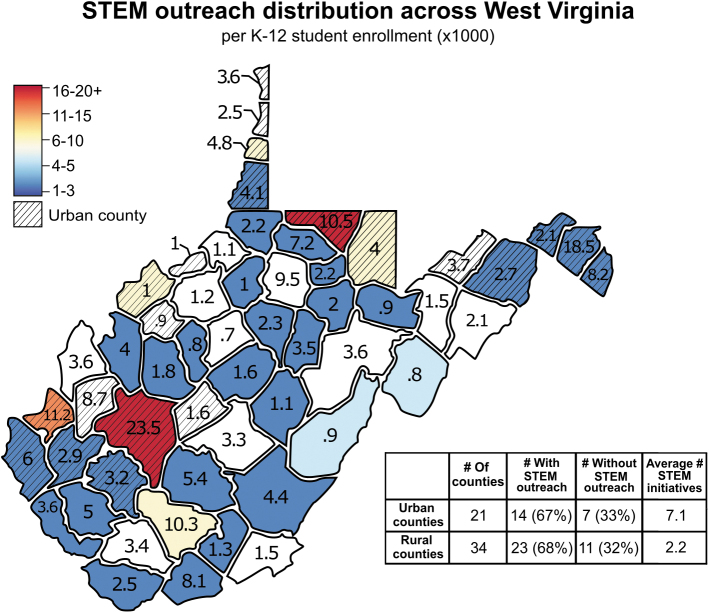
STEM outreach across West Virginia. Map displays the distribution of STEM outreach initiatives (summer camps, science centers, STEM programs, extracurricular clubs, and university-affiliated programs) across counties in West Virginia. The number of K-12 students per county for the 2020–2021 academic year is represented by the value within the county × 1,000 (data from the West Virginia Department of Education). *Cooler colors* indicate fewer outreach initiatives, *warmer colors* indicate more outreach initiatives, and *no color* indicates no outreach initiatives. *Cross hatching* indicates urban counties (data from the Economic Research Service, U.S. Department of Agriculture). Table summarizes the distribution of outreach across rural versus urban counties.

Outreach efforts have grown substantially in West Virginia to foster early interest in STEM. Although there is a generally even distribution of counties that host these initiatives, urban counties contain more of them on average (Fig. 1 and Supplementary Table S1). Of all urban counties with STEM outreach, 50% have three or more initiatives. It is not surprising that outreach is concentrated in populated counties; in Monongalia, home county to West Virginia University, 25 of 37 initiatives are based out of the land grant University. Similarly, 12 of 13 and 9 of 16 outreach initiatives are University affiliated in Kanawha and Cabell counties, respectively, demonstrating how universities play significant roles in facilitating STEM outreach.

Meanwhile, of all rural counties with STEM outreach, 74% have only one or two initiatives, putting students in these counties at disadvantage. Rural students make up almost half of the statewide K-12 enrollment; therefore, this issue affects a significant number of students. It is possible that we are losing many students through the leaky STEM pipeline^3^ due to variable STEM outreach initiative access statewide, and this provides rationale for targeting STEM outreach in underserved counties across West Virginia.

**FIG. 2.**
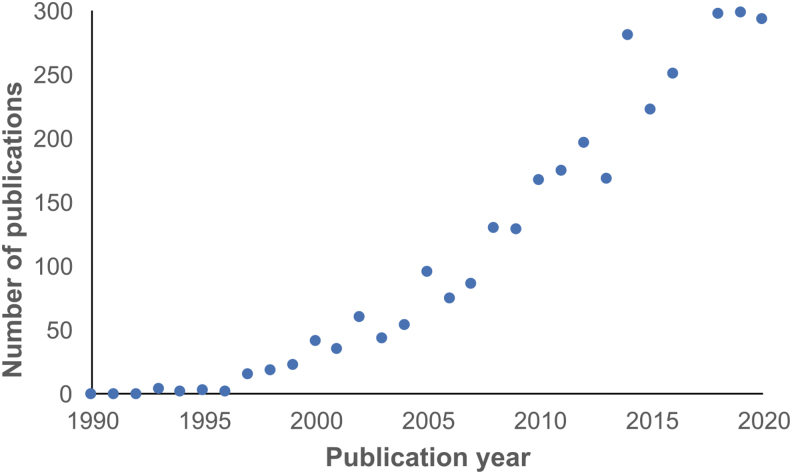
Zebrafish in the education literature. Graph represents the number of publications containing the keywords “zebrafish” and “classroom” from 1990 (0 publications) to 2020 (294 publications). Data obtained from searches conducted on Google Scholar.

Literature describing the use of zebrafish (*Danio rerio*) in classrooms has been increasing steadily since their popularization as a research model (Fig. 2). Zebrafish have many research laboratory benefits that translate well to classroom environments. These benefits include transparent embryos, high reproductive rates, rapid development, and the ability to be housed in relatively small spaces.^22–24^ In addition, zebrafish are genetically and physiologically similar to humans, making them an ideal learning tool for modeling human biology and disease.

Zebrafish have previously been used for teaching topics such as the cell cycle and Mendelian genetics,^25^ toxicology,^26^ body development,^27^ environmental health,^28^ teratogenesis,^29^ motor behavior,^30^ and learning.^31^ Their increasing popularity in STEM education has led to the incorporation of STEM education panels and participation of impacted students at professional science research conferences.^32^

Programs such as Project BioEYES^33,34^ and InSciEdOut^35^ have paved the way for advanced science learning using zebrafish, and they have demonstrated positive shifts in students' content knowledge and attitudes about science. Zebrafish education could be a new avenue for getting students excited about science in West Virginia, and understanding the perceptions of this strategy by students and its feasibility by teachers is key to driving this idea forward.

To integrate enhanced rural science education outreach and using zebrafish in classrooms, we developed a zebrafish research camp for rising high school juniors in West Virginia participating in the Health Sciences and Technology Academy (HSTA).^36^ HSTA is a community-led STEM education program based out of West Virginia University and available to students in 26 counties statewide. Students can apply to the program starting in the 8th grade and participate through high school graduation.

HSTA students each represent at least one of four under-represented student groups: (1) live rurally, (2) first-generation college students-to-be, (3) of low-socioeconomic status, or (4) African American. Since 1998, >3,000 students have graduated from HSTA, with 99% enrolling in college, over half obtaining STEM degrees, and 84% of college graduates remaining in West Virginia to work.^37^ Our partnership with HSTA occurs through their annual biomedical sciences summer camp (BioMed), which is held at West Virginia University.

Our zebrafish camp fits well within the HSTA BioMed curriculum, which places emphasis on engaging in community health. It also fills a gap within the broader scope of STEM outreach in West Virginia, as no established zebrafish outreach education camps exist in-state to our knowledge. Outcomes for students include becoming aware of zebrafish research, practicing relevant laboratory and communication-based research skills, and developing confidence in science capabilities. Protocols for these and other zebrafish educational outreach activities are available by request.

Our first research camp was implemented in 2018, and focused on using zebrafish to answer questions relevant to human and environmental health. In 2019, we incorporated pre–post surveys to assess how our camp impacts students' science confidence. In 2020, our camp was adapted to an online format due to the COVID-19 pandemic, and we used this opportunity to extend our reach to more students and assess general perceptions of online learning, zebrafish, and STEM fields.

During both camps, we surveyed West Virginia teachers participating in HSTA to gauge interest in and feasibility of using zebrafish in their classrooms. Our findings collectively demonstrate success in using zebrafish as a learning tool for under-represented students through in-person or online environments. By keeping top concerns of teachers in mind, future efforts can focus on ways to expand zebrafish education and outreach across West Virginia, especially in counties with minimal STEM outreach opportunities.

## Methods

### In-person camp description

The 2019 camp was held in-person at the WVU Life Sciences Building over the course of 5 days. During the camp, students learned about zebrafish biology, and their applications in biomedicine and environmental health monitoring. Before working with zebrafish, the students practiced important laboratory skills, such as using microscopes and pipetting, as well as theoretical skills, such as using the scientific method and experimental design. We then mentored students through a predesigned experiment that used zebrafish to model fetal alcohol syndrome through ethanol treatments.

Students applied varying concentrations of ethanol (0%, 1%, 2%, and 3%) to fertilized zebrafish embryos (1–2 h postfertilization) in 12-well plates. They predicted outcomes for each treatment and monitored the embryos for 24 h. This guided experiment strengthened valuable skills, including time management, work division, teamwork, and note taking. Next, we mentored students through another experiment that focused on environmental health. We presented a hypothetical scenario based on real-world observations in which we were evaluating the health of local water sources.

We then told the students they would use zebrafish experiments to assess water quality variation in embryonic development. The “water sources” were solutions of egg water (distilled water + Instant Ocean salt at 60 μg/mL final concentration) treated to be varying pH levels. The students discussed potential causes, posed research questions, developed hypotheses, chose parameters to investigate, and recorded data from their zebrafish observations such as heart rate, deformities, and survival.

Results from different student groups were pooled and discussed, and ultimately the students concluded about how zebrafish studies can inform the relationship between environmental and human health. At the conclusion of the week, the students worked together to compose a presentation for all HSTA students, staff, and camp personnel in an end-of-camp symposium. The presentation reviewed camp activities, what they enjoyed the most, the results of their research experiments, and conclusions for how they could apply their camp experiences to local issues in their communities.

### Online camp description

We adapted our camp to an online format in 2020 due to the COVID-19 pandemic. In this format, the students had 2 weeks to complete our zebrafish module as well as other HSTA camp modules. We designed this camp to be asynchronous but still require the critical thinking skills needed to design research experiments. Like the 2019 in-person camp, the 2020 online camp focused on zebrafish biology, their uses in biomedical research, and how they can be used to understand the intersection between human and environmental health. All materials required to complete our module were available through HSTA's online server, and a step-by-step checklist was provided to guide students through recorded presentations, online readings and videos, and subsequent activities.

The required activities taught students about zebrafish biology and their use in research, allowed them to practice experimental design and the scientific method, and guided them in the design of a unique experiment using zebrafish that would solve a hypothetical problem in their community. The activities ranged from online programs and simulators to “Idea Walks” where students went walking in their communities and came up with questions about their environment that could be tested using zebrafish if the students had access to research laboratory facilities and required approvals to carry out the work. At the conclusion of the camp, the students submitted all activities to their HSTA teachers to indicate completion.

### Zebrafish husbandry and ethical approval

All procedures using zebrafish were approved by WVU IACUC. Adult zebrafish (>3 months old) used during HSTA were maintained in the Bergeron Lab fish facility (Department of Biology, WVU). To collect embryos for student observation or experimentation, adult zebrafish were paired in overnight breeding chambers with a divider between them in our fish facility that operates on a 14:10 h light:dark cycle.

The onset of light in the morning stimulates breeding behavior, and spawning occurs once the divider is pulled. Embryos were collected in petri dishes and kept in E3 Embryo Media in a 28.5°C incubator until they were needed for student observation. Before viewing animals, all students took WVU's Occupational Health Questionnaire and completed zebrafish husbandry CITI training.^38^ Students handled embryos only (0–72 h postfertilization), which were humanely euthanized by study personnel after the experiments.

### Student survey design and implementation

Student surveys and parental consent were approved by the WVU Office of Research Integrity and Compliance (ORIC). Surveys were classified as Not Human Subject Research Flex studies (Protocol no. 1904532822). We used an 11-item science confidence questionnaire adapted from previous reports.^39–43^ The questions used a five-point Likert scale ranging from 1 (strongly agree/very confident) to 5 (strongly disagree/not at all confident). The questions reflected major components of student confidence in biology, which are consistent with broad definitions of scientific literacy.

For the 2019 in-person camp, the pre- and postsurveys were administered on paper on the first and final days, respectively. For the 2020 online camp, our surveys were provided through Qualtrics. Students had access to the online presurvey starting 1 week before the camp and completed the postsurvey upon camp completion. In the online survey only, we included an additional section that had uniquely written questions relevant to perceptions of online learning, zebrafish research, and STEM in general. Both the in-person and online surveys collected the students' HSTA identification numbers to allow pre–post results to be aligned and measured while also upholding anonymity.

### Teacher survey design and implementation

Teacher surveys were approved by WVU ORIC and classified as Not Human Subject Research Flex studies (Protocol no. 1904532958). We surveyed teachers using a uniquely developed 3-part questionnaire: Part I collected information pertinent to their teaching (location, subject, class sizes, etc.). Part II assessed desire to implement innovative teaching strategies such as zebrafish in the classroom, and Part III polled for factors or concerns that would prevent them from doing so.

For the 2019 in-person camp, the surveys were administered before the start of HSTA on paper during “teacher training” following a presentation given by study personnel. For the 2020 online camp, the surveys were given through Qualtrics, and teachers completed these before the start of HSTA during an online rendition of teacher training where they watched a prerecorded presentation given by study personnel.

### Analysis of survey data

For both the in-person and online camps, survey responses were organized using Excel. Student responses were first aligned using HSTA identification numbers. To identify trends in responses for a given question across all students, we first identified the number of students selecting each response option (one through five) for that question, and then calculated the percentage of students selecting each option. This was done for pre- and postsurvey responses. To calculate “gain” for each question, the Likert scale was flipped to permit positive gain values (i.e., 1 s were translated to 5 s), and the presurvey responses were subtracted from the postsurvey responses.

We then calculated the average gain and standard deviation of all responses by question and by individual student. A two-tailed *t*-test at *α* = 0.05 was used to identify significant differences in gain on each question between in-person and online students. For the additional questions on the online surveys that collected student perceptions, as well as for the teacher survey data, similar methods were used to calculate the average response or percentage of participants selecting a response on a given question. A two-tailed *t*-test at *α* = 0.05 was also used in the teacher surveys to identify significant differences in response selection between rural and urban teachers.

### West Virginia STEM outreach initiative data

Online research was conducted to compile a list of STEM outreach initiatives across West Virginia by county. First, webpages for 4-year institutions, 2-year degree programs, and community colleges were searched for departments, programs, clubs, or other initiatives that provide STEM experiences to students in the county. Then, general searches using keywords such as “STEM” and “outreach” combined with each county name were conducted to identify other initiatives such as community outreach programs, summer camps, clubs, and science centers.

If a single initiative was dispersed across several parts of a county (such as after-school programs occurring in many schools), each school was counted as a single initiative. In addition, K-12 student enrollment data for the 2020–2021 year were obtained from the West Virginia Department of Education website, and rural or urban status for each county was obtained from the Economic Research Service website (United States Department of Agriculture).

## Results

### Student survey population

During our 2019 in-person year, 12 students participated in our camp and surveys. All students belonged to more than one under-represented student group as defined by HSTA; 33% were African American, 50% were of low-socioeconomic status, 92% were first-generation college students-to-be, and 100% lived rurally. In addition, 58% of students identified as male and 42% identified as female.

During our 2020 online year, 83 students participated in the virtual zebrafish camp. Sixty-five successfully completed both the pre- and postsurveys (78% response rate). Thirty-seven percent of these students were African American, 41% were of low-socioeconomic status, 51% were first-generation college students-to-be, and 70% lived rurally. Sixty-four percent of students fell into more than one of these categories. In addition, 61% of students identified as female, 27% identified as male, and 12% did not wish to specify.

### Teacher survey population

During our 2019 in-person year, 13 teachers participated in our surveys, and seven of them were science teachers. These teachers represented 9 of 55 WV counties; 6 were from rural counties, and 7 were from urban counties. These teachers reported overseeing a range of 100–180 students in a single academic year. During our 2020 online year, 42 of 65 teachers in HSTA participated in our virtual teacher training and surveys (64% response rate); 22 of 42 were science teachers. These teachers represented 24 of 55 WV counties; 21 were from rural counties, and 21 were from urban counties. The nine counties represented in the in-person year were also represented in the virtual year.

Fifteen counties were newly represented virtually; nine of these were rural, and six were urban. These teachers reported overseeing a range of 30–180 students in a single academic year. The range of total students taught yearly was more variable with much smaller student totals in the virtual survey compared with the in-person survey, and these low numbers were reported from teachers working in two rural counties. Overall, our reach to rural and urban teachers is mostly evenly distributed; however, we did reach slightly more rural counties in our online year.

### Students demonstrate increased science confidence after the zebrafish camp

**FIG. 3.**
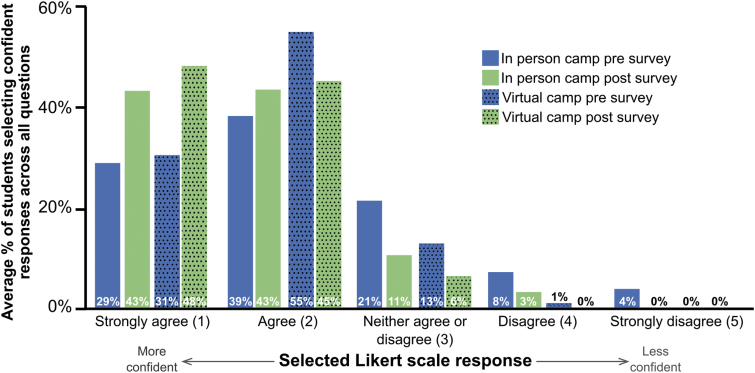
Students report higher science confidence after the zebrafish camp in both in-person and online formats. Graphs represent average student responses across all questions for the pre (*blue bars*) and post (*green bars*) surveys. *Solid bars* represent data from our 2019 in-person camp (*n* = 12 students), and *dotted bars* represent data from our 2020 online camp (*n* = 65 students). Answers from students were ranked on a Likert scale ranging from 1 (strongly agree) to 5 (strongly disagree).

Participating in hands-on research experiences has been shown to positively impact students' science confidence.^44–47^ To assess how our zebrafish camp impacts science confidence, we implemented pre- and postsurveys adapted from previous reports.^39–41^ Between the in-person and online camps, we observed similar positive shifts in confidence averaged across all questions from pre to post (Fig. 3 and Supplementary Table S2), indicating that students left feeling more confident in their science skills.

We also saw that over half of our students were already somewhat confident in their science skills coming into the camp; 68% and 86% of students on average selected confident presurvey values (i.e., one or two) across all questions in the in-person and online camps, respectively. For both camps, the largest confidence gain was on Q1, which asked about confidence in composing the introduction to a laboratory report (Table 1; in-person gain = 1.00, online gain = 0.48). However, these gains are also significantly different between the camps (*p* = 0.03), indicating that confidence gain was significantly lower in the online camp. Since this was the largest gain observed for the online camp, we can infer that gains in science confidence were lower across the board compared with the in-person camp.

**Table 1.** Calculated Gains in Science Confidence Between the In-Person and Online Camp

Survey question	In-person camp average (±SD)	Online camp average (±SD)
1. I am confident that I could write an introduction to a laboratory report or presentation.^*^	1.00 (0.91)^*^	0.48 (0.70)^*^
2. I am confident that I could write the methods section and describe experimental procedures of a laboratory report or presentation.	0.58 (0.49)	0.45 (0.72)
3. I am confident that I could write up the results to a laboratory report or presentation.	0.42 (1.04)	0.26 (0.61)
4. I am confident that I could write the conclusion to a laboratory report or presentation.	0.58 (1.04)	0.26 (0.66)
5. I am confident that I could read the procedures for an experiment and feel sure about conducting the experiment on my own.	0.33 (0.62)	0.25 (0.68)
6. I am confident that I will be successful in a biology course.	0.33 (0.62)	0.18 (0.78)
7. I am confident that I could explain something that I learned at HSTA to another person.	0.00 (0.41)	0.17 (0.71)
8. I am confident that I could use the scientific method to solve a problem.	0.25 (0.92)	0.22 (0.69)
9. I am confident that after reading an article about a biology experiment, I could write a summary of its main points.	0.67 (1.11)	0.32 (0.61)
10. I am confident that after reading an article about a biology experiment, I could explain its main ideas to another person.	0.25 (1.30)	0.25 (0.77)
11. I am confident in my abilities to work with a group.^*^	0.58 (0.86)^*^	0.18 (0.55)^*^

Table represents the average gain in science confidence for all questions for the in-person (*n* = 12) and online (*n* = 65) zebrafish camps. Answers from students were ranked on a Likert scale ranging from 1 (strongly agree) to 5 (strongly disagree). Questions and gain values with asterisks (^*^) are significantly different between the camps as determined by a two-tailed *t*-test at *α* = 0.05.

HSTA, Health Sciences and Technology Academy; SD, standard deviation.

For both camps, the smallest gain was on Q7, which asked about confidence in explaining a topic learned at HSTA to another person (Table 1; in-person gain = 0.00, online gain = 0.17). The largest gap in gain between both camps was on Q11, which asked about confidence in working with a group. The online camp gain (0.18) was significantly lower than the in-person camp gain (0.58, *p* = 0.04), which is not surprising due to the nature of online environments making groupwork feel less authentic.

In general, positive shifts in confidence were most noticeable on questions related to performing scientific tasks independently (Qs 1, 2, 3, 9) and less so on questions relevant to the students' perceptions of their own ability to be successful (Qs 6, 8, 10, 11). These results suggest that students perceive their ability to write about science more strongly than their ability to succeed in a collaborative workspace or apply their knowledge to a new problem. The lower gains in the latter criteria could reflect inherent reservations to participate in these activities, which would agree with reports describing lower STEM success expectations by minority and under-represented students.^48–51^

### Perceptions of online learning, zebrafish, and STEM fields

We included additional questions on the postsurvey for our 2020 online year that focused on three topic areas: online learning, zebrafish, and STEM research in general (Table 2). The first topic area, online learning, has become increasingly relevant in educational practices, offering flexibility and independence more so than traditional environments.^52–54^ However, integrating laboratory-based practices that use real experimental methods and data into online spaces proves difficult. For the rural students in this study, there are additional challenges that complicate their ability to participate in online learning, such as poor internet connection.

**Table 2.** Perceptions About Online Learning, Zebrafish, and STEM

	Average (±SD)	% agree or strongly agree
Questions about online learning		
1. In general, I enjoy learning online.	2.60 (0.94)	50
2. Being able to learn at my own pace allowed me to understand concepts better/at a deeper level.	1.98 (0.91)	80
3. I enjoyed attending the virtual zebrafish camp at HSTA BioMed.	2.01 (0.66)	78
Questions about zebrafish		
4. Zebrafish camp content was beneficial to my understanding of the biomedical sciences and research in general.	1.81 (0.70)	86
5. I would enjoy performing the experiment I designed using zebrafish in a research laboratory.	1.72 (0.69)	87
6. I enjoyed learning about zebrafish and their uses in biomedical research.	1.67 (0.61)	93
7. I would enjoy learning using zebrafish or other hands-on model systems in school.	1.60 (0.56)	96
8. I would enjoy learning more about other animal models used for research.	1.67 (0.59)	94
Questions about STEM/research in general		
9. I enjoyed using the scientific method to solve a theoretical question in my community/household.	2.00 (0.68)	77
10. The zebrafish camp has made me more interested in conducting research.	2.01 (0.79)	74
11. Attending the zebrafish camp has made me more interested in applying for a STEM college degree.	2.10 (0.79)	67

Table represents the average response from students participating in the online zebrafish camp only. Answers from students were ranked on a Likert scale ranging from 1 (strongly agree) to 5 (strongly disagree). “% Agree or Strongly Agree” represents the percentage of students (total *n* = 65) agreeing with the statement (i.e., selecting one or two).

We found that only half of our students agreed to finding online learning enjoyable (Q1). However, the majority of students agreed that online environments allow them to learn at their own pace and better understand concepts (Q2), and they also agreed that the online zebrafish camp was enjoyable (Q3). This suggests that negative perceptions toward online learning exist, which could be addressed with increased development of accessible, well-designed online experiences. A strong interest in zebrafish was quite evident in the second topic area, with nearly all students agreeing that they would enjoy learning more about zebrafish in school (Q4–7).

Finally, in the third topic area, we found that our camp inspired interest in research and STEM degrees. Most students enjoyed using the scientific method to solve problems (Q9), and they agreed that they felt more interested in pursuing research after our camp (Q10). In addition, over half of our participants agreed that they ended the camp feeling more interested in STEM degrees (Q11). Collectively, these results suggest that future learning experiences using zebrafish would be received well by students. Our methods show that zebrafish learning experiences can be applied to online spaces in an enjoyable and educational way, which is an important consideration for future online STEM educational outreach development.

### West Virginia teachers experience challenges related to supplies, experience, and funding

**FIG. 4.**
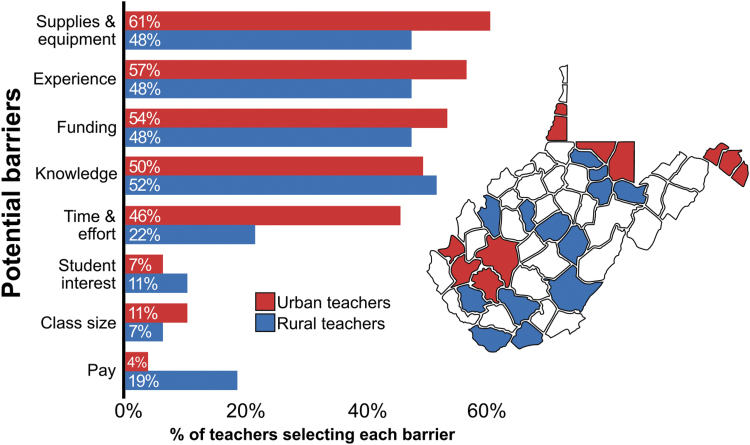
Concerns for implementing new approaches to science education as reported by HSTA teachers. Graph represents the percentage of teachers (total *n* = 55, rural *n* = 27, urban *n* = 28) selecting factors that might prevent them from using zebrafish or other innovative strategies as a tool for enhanced science education. *Red bars* represent urban teachers, and *blue bars* represent rural teachers. Map depicts the distribution of counties represented by the teachers (*n* = 24/55 counties). The color code corresponds to the color code on the graph; *red* counties are urban and *blue* counties are rural. HSTA, Health Sciences and Technology Academy.

Previous reports have successfully used zebrafish for experiments in classroom settings.^25,28,33,35,55^ However, teachers may face various challenges that prevent them from implementing this strategy.^7–11^ To better understand which factors are most concerning for teachers in West Virginia, we surveyed teachers participating in HSTA. We found that the top three concerns among urban and rural teachers are availability of supplies and equipment, experience, and funding (Fig. 4 and Supplementary Table S3). Selection of these factors by rural and urban teachers was generally even. Supplies and equipment was a top concern for both groups of teachers, suggesting that similar constraints exist regardless of rural or urban status.

Two factors with variable responses across rural and urban teachers were time and effort, which was selected more by urban teachers, and pay, which was selected more by rural teachers. As previously mentioned, some teachers from urban counties reported being responsible for a larger range of students annually compared with rural teachers. Therefore, these teachers may perceive integrating zebrafish into their curricula as less feasible because of high student volume creating limited time.

Pay is a pervasive issue within rural education in general,^7–11^ so it is not surprising that this would discourage some rural teachers from using zebrafish. Overall, these surveys provide insight from teachers working in 24 of 55 West Virginia counties, 11 being urban and 13 being rural. As our distribution of teachers from rural versus urban counties is comparable, we believe that these data are representative of the challenges faced by teachers statewide.

### West Virginia teachers are interested in using zebrafish but require varied support

The majority of teachers agreed that zebrafish would get their students excited about science (Table 3). However, we observed mixed opinions regarding feasibility and desire for implementing zebrafish into classrooms. Familiarity with zebrafish by teachers in West Virginia is minimal, and as a result, only 30% and 25% of rural and urban teachers agreed that they would be willing to host a zebrafish tank in their classroom, respectively. However, almost half of all teachers agreed that they would be willing to pursue this strategy if they were provided training, guidance, and comprehensive manuals for care and experimentation.

**Table 3.** Insights on Feasibility and Desire for Incorporating Zebrafish into Science Classrooms as Reported by Health Sciences and Technology Academy Teachers

Survey question	Rural teacher average (±SD)	Rural teachers: % agree or strongly agree	Urban teacher average (±SD)	Urban teachers: % agree or strongly agree
1. I have access to external funding, such as grants, in my school.	2.89 (0.97)	44	2.46 (0.74)	61
2. I have access to internal school funding in my school.	2.42 (0.99)	63	2.50 (0.96)	61
3. I have access to microscopes and other science equipment in my school.	2.19 (0.85)	67	1.93 (0.86)	82
4. I have used animals in my classroom before.	3.50 (1.53)	33	3.43 (1.4)	25
5. I have flexibility in my teaching regimen to incorporate new techniques.	2.12 (1.07)	74	2.11 (1.07)	82
6. Students at my school have access to extracurricular science outreach/education events.	2.59 (1.12)	44	2.25 (0.65)	64
7. I would be able to get permission to install a zebrafish tank in my classroom.	2.19 (1.17)	67	2.57 (1.17)	54
8. I am familiar with aquatic system husbandry.	3.46 (1.24)	22	3.50 (1.35)	25
9. I am familiar with zebrafish biology, husbandry, & education.	3.96 (1.15)	15	3.79 (0.99)	7
10. I am likely to incorporate zebrafish into my science curriculum based on my current familiarity.	3.28 (1.43)	30	3.38 (1.13)	25
11. I am likely to incorporate zebrafish into my science curriculum after receiving training and guidance.	2.40 (1.19)	56	2.85 (1.19)	43
12. I believe zebrafish might help excite students and aid learning in science.	1.96 (1.09)	81	1.86 (0.65)	86
13. I am interested in incorporating a new method of science education in my classroom.	2.12 (0.88)	67	2.44 (1.05)	54
14. I am more likely to have a science outreach program visit my class than to install a zebrafish tank and conduct my own experiments with students.	2.84 (0.99)	33	2.63 (1.15)	43
15. If a comprehensive zebrafish care and use guide was provided to me, I would be more interested in using zebrafish in my classroom.	2.12 (0.99)	78	2.70 (1.14)	43

Table represents average responses from HSTA teachers (total *n* = 55, rural *n* = 27, urban *n* = 28) indicating their agreement with statements relevant to their teaching. Answers from teachers were ranked on a Likert scale ranging from 1 (strongly agree) to 5 (strongly disagree). “% Agree or Strongly Agree” represents the percentage of teachers agreeing with the statement (i.e., selecting one or two). No significant differences exist between rural and urban teacher responses as determined by a two-tailed *t*-test at *α* = 0.05.

Although no significant differences were observed between rural and urban teacher responses, there is some variability across some of the survey questions. On average, less rural teachers agreed with Qs 1, 3, and 6. These questions are related to external funding availability, access to adequate science equipment, and presence of science outreach and extracurriculars for students, all of which are pervasive issues within rural STEM education.

On the contrary, a larger percentage of rural teachers agreed with Q15, which was related to willingness to implement zebrafish into their classroom. It is interesting to note that despite their identified challenges, rural teachers are still interested in this strategy. Overall, these varied responses indicate that feasibility and desire to implement zebrafish into classrooms depend on diverse factors. Zebrafish provide an avenue for student learning and experimentation that these teachers are clearly interested in; however, individual collaborations would be necessary to support specific teacher needs.

## Discussion

### Zebrafish research experiences yield positive outcomes for under-represented students

Our zebrafish research experiences positively impact science confidence among under-represented students in West Virginia (Fig. 3 and Table 1). A strong interest in zebrafish, research, and STEM degrees is also evident based on our surveys (Table 2). This work provides rationale for continued development of zebrafish learning experiences in the state, whether in-person or virtual. Efforts like these in West Virginia and beyond could positively influence student perceptions of STEM, which is particularly important for under-represented and minority students. Lower interest in STEM has been reported to appear early in these student groups, and it results from a combination of factors.

Some factors include inadequate funding and limited resources for education development in their schools, negative perceptions of STEM, low expectations of their own success, family and peer influence, and inadequate educational and career support through high school.^48–51^ HSTA students must qualify for at least one of four under-represented student groups, and these under-represented groups have been specifically associated with lower performance and interest in STEM.^56–63^ Thus, it is important to reach under-represented students early with engaging STEM opportunities that are shown to work, such as implementing zebrafish in ways that model our approaches.

### Success within partnerships with HSTA

Our work demonstrates the success and effectiveness of a zebrafish research camp in a largely rural state made possible through the partnership of a zebrafish research laboratory and university-affiliated outreach organization. HSTA describes their model as one for “brain gain” that is ready to be replicated in any state in the nation,^64^ thus the development of similar initiatives could foster widespread interest in STEM. For others interested in developing their own zebrafish education initiatives similar to ours, partnering with an existing outreach organization is a great method for establishing the basis of a zebrafish research camp.

By forming these partnerships, zebrafish laboratories at any institution in any state can extend their reach beyond research and get students excited about science. In addition, important data can be collected using our methodology that will inform the continued growth of the program. Although our positive results are exciting, these data only represent two cohorts of HSTA students. HSTA follows students through college graduation, and of interest would be following up with zebrafish camp alumni to collect information on major declaration, status in research, and if they believe the zebrafish camp had any influence on decisions made during their academic career.

### The growing era of online learning

Students participating in our online zebrafish camp expressed strong interest in camp content, zebrafish, and research in general without a hands-on experience. These results demonstrate that online experiences can foster positive outcomes similar to in-lab experiences. Likewise, the onset of a global pandemic in 2020 necessitates that online learning tools and modules like ours are developed. There are currently several virtual platforms that simulate in-lab research experiences, such as Labster^65^ and PraxiLabs.^66^

Virtual zebrafish research experiences specifically could become an opportunity for students who otherwise have limited access to an in-lab experience. Several online databases and resources exist for learning about zebrafish,^67,68^ and zebrafish even made their debut on mobile platforms in an interactive app called SimUFish that focuses on modeling behavior.^69^ However, the development of online experiences is complicated by issues in accessibility. Online learning strategies exclude certain groups of students, such as students living rurally with limited internet access.

In West Virginia, it is estimated that many students lack sufficient internet access or speed to participate in online activities, and some do not own or have not been provided devices for distance learning.^70^ Progress must be made on these issues for online STEM outreach to be accessible for all students, and our work supports the need for this progress to be made.

### Supporting West Virginia teachers in integrating zebrafish learning into science curricula

HSTA is an optimal medium for introducing students to zebrafish research. One avenue for growing programs like ours is direct collaborations with teachers. Partnerships between researchers and high school teachers have previously been successful, and these reports offer a framework for mirroring them.^25,71^

We suggest beginning partnerships with in-lab training experiences to provide the teachers with initial exposure to working with zebrafish. Then, specific protocols and support guides can be developed as a resource for these teachers. Specifically, we have produced a comprehensive zebrafish care and experimentation pamphlet to assist teachers through a fish tank setup process. Collaborations between research laboratories and teachers in West Virginia is feasible, but support tailored to each situation is required. Partnerships in West Virginia will ultimately permit growth beyond HSTA and allow more students to experience learning using zebrafish.

Most of our teachers agreed that they are interested in incorporating new teaching strategies into their science curricula. However, almost half were more likely to recruit an outreach team rather than pursue using zebrafish themselves. As such, another avenue for growing outreach efforts like ours is to follow models set by initiatives such as Project BioEYES and InSciEdOut. With proper funding, personnel, and interest, our zebrafish research camp could become an outreach program that extends beyond just HSTA and is available to any classroom in West Virginia.

For our case and beyond, success in collaborations like these is dependent on teachers having access to experimental protocols and modules to implement. Increased development of materials that are accessible and feasible for teachers, as well as consistent support from researchers, is essential for zebrafish to become a more prominent educational tool used by K-12 teachers.

## Conclusion

In this report, we highlight success in developing and implementing a zebrafish research camp in both in-person and online formats. Our methods can be modeled by others developing zebrafish research experiences in any state. Based on our findings, we believe that zebrafish research experiences, whether in-person or virtual, will yield significant positive outcomes for diverse students, but particularly for under-represented students who otherwise would not have access to such an experience.

Likewise, teachers in West Virginia believe that zebrafish would excite their students about science, however require varied support based on their situations. Future efforts will focus on continuing our partnership with HSTA, and determining methods for extending our reach beyond our program through direct and now largely informed partnerships with teachers that are based on their needs.

## Supplementary Material

Supplemental data

Supplemental data

Supplemental data
